# Physiological joint line total knee arthroplasty designs are especially sensitive to rotational placement – A finite element analysis

**DOI:** 10.1371/journal.pone.0192225

**Published:** 2018-02-05

**Authors:** Philippe Moewis, Sara Checa, Ines Kutzner, Hagen Hommel, Georg N. Duda

**Affiliations:** 1 Julius Wolff Institute, Charité-Universitätsmedizin Berlin, Berlin, Germany; 2 Krankenhaus Märkisch-Oderland GmbH, Klinik für Orthopädie und Unfallchirurgie, Strausberg, Germany; University of Memphis, UNITED STATES

## Abstract

Mechanical and kinematical aligning techniques are the usual positioning methods during total knee arthroplasty. However, alteration of the physiological joint line and unbalanced medio-lateral load distribution are considered disadvantages in the mechanical and kinematical techniques, respectively. The aim of this study was to analyse the influence of the joint line on the strain and stress distributions in an implanted knee and their sensitivity to rotational mal-alignment. Finite element calculations were conducted to analyse the stresses in the PE-Inlay and the mechanical strains at the bone side of the tibia component-tibia bone interface during normal positioning of the components and internal and external mal-rotation of the tibial component. Two designs were included, a horizontal and a physiological implant. The loading conditions are based on internal knee joint loads during walking. A medialization of the stresses on the PE-Inlay was observed in the physiological implant in a normal position, accompanied by higher stresses in the mal-rotated positions. Within the tibia component-tibia bone interface, similar strain distributions were observed in both implant geometries in the normal position. However, a medialization of the strains was observed in the physiological implant in both mal-rotated conditions with greater bone volume affected by higher strains. Although evident changes due to mal-rotation were observed, the stresses do not suggest a local plastic deformation of the PE-Inlay. The strains values within most of the tibia component-tibia bone interface were in the physiological strain zone and no significant bone changes would be expected. The physiological cut on the articular aspect showed no detrimental effect compared to the horizontal implant.

## Introduction

Over the last decades, total knee arthroplasty (TKA) has proved to be a successful procedure for the treatment of patients with severe osteoarthritis of the knee joint with survival rates of 95% at 15 years reported [1, 2]. However, around 20% of patients are dissatisfied with the outcome, citing limited function, residual pain and instability as the main reasons [3]. Related to this dissatisfaction is the optimal alignment of the implant components, which remains a matter of debate [4].

Traditionally, mechanical alignment is the usual procedure for a TKA component alignment, where a horizontal neutral lower limb axis is created by cutting the distal femoral and proximal tibia bone at 90° respect to the mechanical axis [4]. This procedure aims for a more homogeneous medio-lateral force distribution, which is considered an important factor related to the durability of the implant [5–8] and also to be relevant for patient function [9, 10]. However, this cut perpendicular to the lower limb axis leads to an alteration of the native joint line orientation of the patient and a rather large bony resection [11–14].

The concept of kinematical alignment has been discussed as an alternative to mechanical alignment [8, 15–17]. In this procedure, the femoral and tibial components are positioned in such a way that the natural angle and level of the distal and posterior joint lines are preserved and the patient´s natural anatomy of the knee kept [11, 18, 19]. Since 98% of normal limbs are not straight [20] and 32% of men and 17% of women have an alignment of 3° varus, the so called “constitutional” varus [18], it could be assumed that a relevant portion of the population could benefit from the resulting physiological joint line instead of a perpendicular joint line alignment.

However, a recent study conducted by Young and colleagues [21] could not identify specific advantages in the patient´s reported outcome after two years with the kinematical alignment technique and recommended this procedure to be used with caution due to the unknown effects on implant durability.

Regardless of the alignment procedure, a major challenge might be the influence of an axial mal-rotation of the tibia component, since it is known to be one of the most common procedure errors during TKA [22]. Such mal-placement could lead to an increase in the contact pressure on the articular polyethylene surfaces [23, 24] which might increase the risk of fracture and wear of the polyethylene inlay [25] or lead to abnormal load transfer to the underlying/supporting bone [26].

A specially designed implant that emulates the physiological joint line could help to overcome the patient´s specific alignment problems. On the other hand, such special implant geometry could lead to mechanical overload in the polyethylene (PE) inlay and/or the bone side of the tibia component-tibia bone interface if mal-rotated.

The aim of this study was to determine the influence of a total knee prosthesis with a physiological joint line on the distribution of stresses in the inlay as well as the load transfer to the tibia component-tibia bone interface after normal and mal-rotated implantation compared to a conventional total knee design.

## Materials and methods

Finite element (FE) calculations were conducted to analyse the stress distribution on the PE-Inlay, as well as the mechanical strain distribution at the tibia component-tibia bone interface.

Two TKA implants were included, a traditional horizontal implant (fixed bearing, cruciate retaining, 4° tibial posterior slope) and an implant with an physiological joint line (fixed bearing, cruciate retaining, 3° varus inclination, 4° tibial posterior slope); both geometries were developed by OHST AG, Rathenow, Germany.

**Fig 1:**
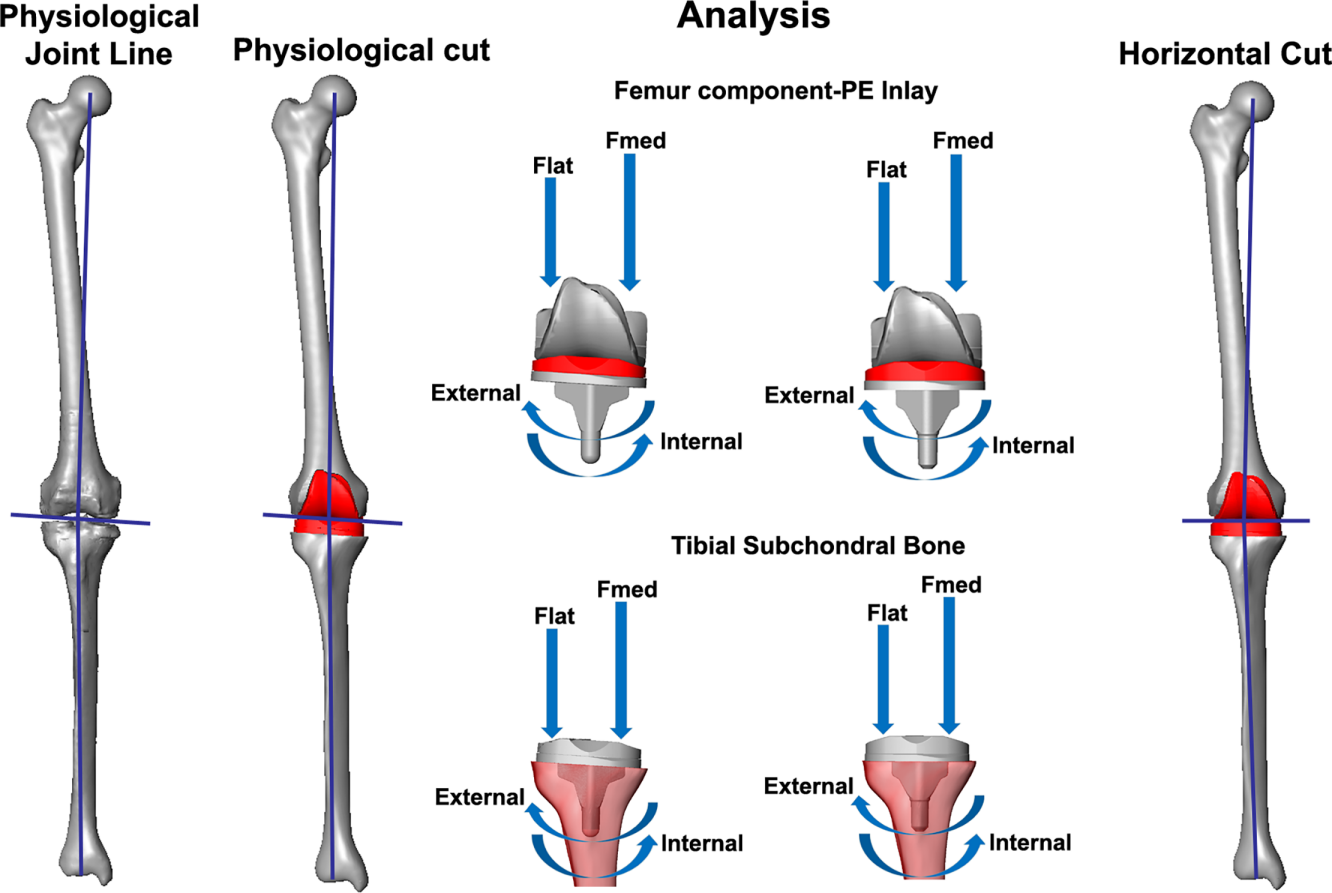
Description of the overall analysis.

The following conditions were analysed: normal positioning of both femoral and tibial components, 5° and 10° axial external mal-rotation of the tibia component and 5° and 10° axial internal mal-rotation of the tibia component (Fig 1), these values were considered after discussion with our knee surgeon and also based in axial rotation values ranging from 0.4°-13.2° reported by Berger et al. 1998, Barrack et al. 2001, Bedard et al. 2010 [27–29]. The positioning of the femoral and tibial components was also supervised by our knee surgeon. The patella was not considered for the analysis.

### Analysis of the stress distribution on the PE- Inlay

#### Construction of the solid models and material properties definition

CAD surfaces of the described implants were imported into Abaqus FEA and meshed with tetrahedral elements (standard quadratic, Abaqus element numbers: 146690, 22015 and 16325 for the PE inlay, tibia and femur components respectively). The “characteristic element length” of the models is between 1–3.4mm fulfilling the criteria for enough convergence according to Viceconti et al. 2005 and Cristofolini et al. 2010 [30, 31].

The femur and tibia components of both physiological and horizontal implants are made of Cobalt-Chrome-Molybdenum Alloy (CoCrMo, ISO5832-4): E = 210000 MPa, ν = 0.3. Both PE inlays are made of Ultra High Molecular Weight Polyethylene (UHMWPE, ISO5834-2): E = 1200MPa, ν = 0.46, yield stress 14.07MPa. All parts were defined as linear (due to the small deformations/displacements expected) elastic-plastic, homogeneous materials.

#### Boundary conditions

Postoperative computer tomography (CT) images of a patient with a telemetric implant [32] (male, 66 years, 100 Kg) were used as a guide to guarantee a proper positioning of the femoral and tibia components.

For the contact definition between the femoral component and the PE inlay, the Normal Behavior was modelled as an exponential contact pressure overclosure relationship, default constraint enforcement method. For the Tangential Behavior the friction formulation was chosen as penalty. The friction coefficient was defined as 0.07. The analysis performed was an implicit linear calculation. Due to the small displacements expected, NLgeom was set off. Two steps were defined for the calculation, starting with a vertical movement in the longitudinal direction until first contact between the femoral component condyles and the PE inlay was reached, then a free movement of the femoral component was permitted. The lower face of the PE inlay was fixed during the whole analysis. Ligament forces were considered negligible and therefore not included in the model due to the known high stability of the knee joint in the stance phase, which is achieved mostly by muscle contraction and body weight [33].

#### Loading conditions

Previously measured loads during walking of a patient with a telemetric implant (INNEX Fixuc, Zimmer Biomet, implanted in a mechanical alignment procedure) were used for the definition of the loading conditions in all the analyses. During walking, two main force peaks occurred at the instant of contralateral toe off and shortly before contralateral heel strike [34]. The loads associated to these instants (particularly the second peak) were used as the reference for the calculation of the medio-lateral axial load distribution according to Halder and colleagues [35]. Here, the measured axial force (Fz) as well as the adduction moment (My) was used to calculate the specific medial (Fmed) and lateral (Flat) forces, which resulted in a distribution of approximately 60% vertical load (1270 N) for the medial compartment and 40% vertical load (902 N) for the lateral compartment (Fig 1). The calculated forces were correspondingly applied in the medial and lateral compartments. Additionally, the same analysis was also conducted with a homogeneous medio-lateral distribution of 50% vertical load (1086 N) medial and 50% vertical load (1086 N) lateral as well as 70% vertical load (1520 N) medial and 30% vertical load (652 N) lateral.

### Analysis of the mechanical strains distribution at the tibia component-tibia bone interface

#### Construction of the solid models and material properties definition

Two three dimensional (3D) solid models of the tibia bone were reconstructed from preoperative CT scans of the aforementioned patient using the commercial softwares Amira (Zuse Institute Berlin, Berlin, Germany) and Geomagic (Geomagic Inc. Morrisville, North Carolina, USA), then imported into Abaqus FEA and meshed with tetrahedral elements (standard quadratic, Abaqus element number: 399265). The “characteristic element length” of the models is between 1–3.4mm fulfilling the criteria for enough convergence according to Viceconti et al. 2005 and Cristofolini et al. 2010 [30, 31]. Both tibia bone models were divided, using the CT scans as reference, into cortical bone (E = 17000 MPa, ν = 0.33) and cancellous bone (E = 1000 MPa, ν = 0.33) and defined as linear elastic, homogeneous material, based on the reports by Juszczyk et al. 2011 [36]

#### Boundary conditions

After positioning of the tibia components, the most distal face of both tibia component plateaus were used to define two cutting planes to remove the articular surface of the tibia bone, leading to a 3° inclined (physiological) and a horizontal cut respectively. The keels and the shaft of both tibial components were used to perform a cut of the tibia bone.

A press fit implantation was modelled with a hard contact defined at the tibia component-tibia bone interface with a friction coefficient of 0.5. In order to simplify computational calculations, the tibia bone was cutted at approximately 20 cm below the joint line. A fixed boundary condition (encastre) was defined at the more distal portion of the tibia shaft while the tibia component could move freely inside the tibia bone only restricted by the defined hard contact. As mentioned in the previous model description, this selected boundary condition and not a definition of ligaments was chosen due to the fact that the applied loads correspond to the moment in the stance phase were the knee joint is almost at full extension, which is known to be an instant of high stability due to muscle contraction and body weight [33].

#### Loading conditions

The aforementioned loading scenario was also used for the strains distribution analysis at the tibia component-tibia bone interface (Fig 1). The two force vectors were applied directly on the tibia plateau.

The described model specifications were also applied to the mal-rotation models, with the addition of external and internal axial mal-rotation of the tibial component, respectively.

To properly quantify the influence of the strains determined, the proximal portion of the tibia bone was selected as region of interest (considering all the contact area between tibia component and tibia bone) and the strains of each element in this volume in all conditions were plotted in a bar chart.

## Results

### PE inlay

**Fig 2:**
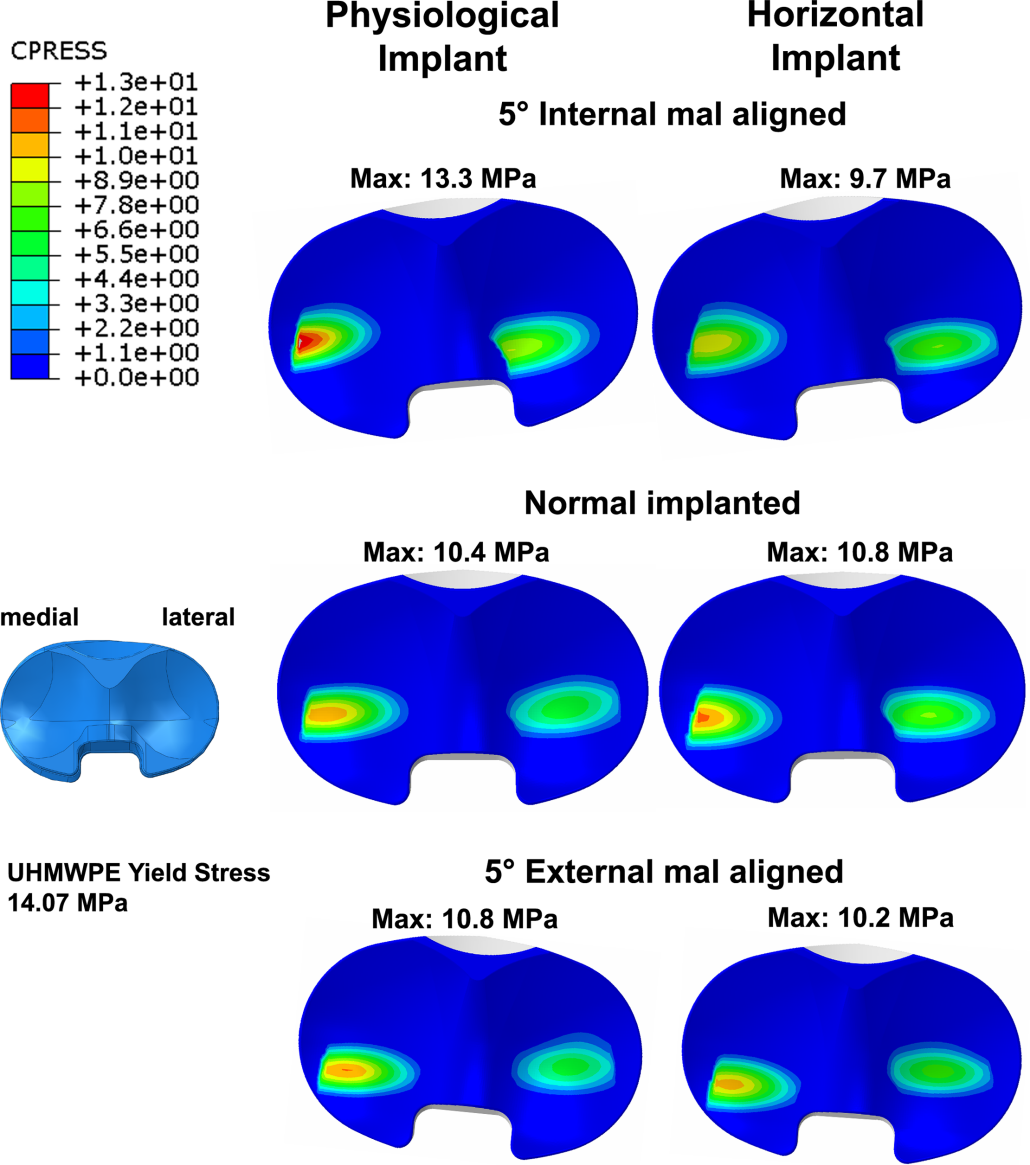
Contact pressures (MPa, 60/40 medio-lateral load distribution) on the PE-inlay of implants with a physiological joint line and a horizontal line. Contact pressures are calculated in a normally positioned implant, as well as under 5 degrees of internal and external mal-rotation of the tibial component.

Assessment of the contact pressure on the PE-Inlay with a normal positioned tibia component under all the aforementioned medio-lateral loading conditions resulted in a medialization of the contact pressures in the physiological and the horizontal implants, an example for the 60/40 medio-lateral distribution can be observed in Fig 2.

Considering the mal-rotated conditions, a more pronounced medialization of the contact pressures was observed at 5° and 10° internal mal-rotation in all the analysed loading conditions in the physiological implant compared to the horizontal implant.

The same medialization pattern could be observed at both 5° and 10° rotations during the external mal-rotation analysis. All the values are collected in Table 1.

**Table 1:** Contact pressures (CPRESS) in the medial and lateral compartments for all the loading conditions as well as in normal and mal-aligned implantation.

CPRESS (MPa)	Normal		5° Internal		5° External		10° Internal		10° External	
	Medial	Lateral	Medial	Lateral	Medial	Lateral	Medial	Lateral	Medial	Lateral
**Phy. (50/50)**	9.5	7.1	11.6	9.2	9.7	6.9	11.9	10.5	10.4	6.8
**Hor. (50/50)**	9.6	7.8	8.6	8.5	8.6	7.9	8.8	8.6	8.6	7.8
**Phy. (60/40)**	10.4	6.2	13.3	8.6	10.8	6.1	13.3	8.9	11.6	6.0
**Hor. (60/40)**	10.8	6.7	9.7	7.6	10.2	7.1	10.1	7.7	9.6	6.6
**Phy. (70/30)**	12.3	5.2	15.6	7.9	12.5	4.9	15.8	8.3	13	5.1
**Hor. (70/30)**	12.5	5.4	11.3	6.3	10.9	5.4	11.9	6.4	11	5.3

### Tibia component-tibia bone interface

A homogeneous strain distribution in the tibia component-tibia bone interface can be observed using both implant geometries in the normal implanted condition. On the other hand, a clear medialization of the strains can be observed in the physiological inclined implant in both internal and external mal-rotated conditions. Particularly during the externally mal-rotated condition the higher strains were mostly concentrated in the posterior-medial zone. This behaviour was also similar in the horizontal implant during the externally mal-rotated condition.

Most of the interface volume presented strains in the physiological zone according to Basso and colleagues [37]. Less than 1% of the volume in all the conditions analysed showed a range of strains between 2500 and 5000 με (overused zone).

**Fig 3:**
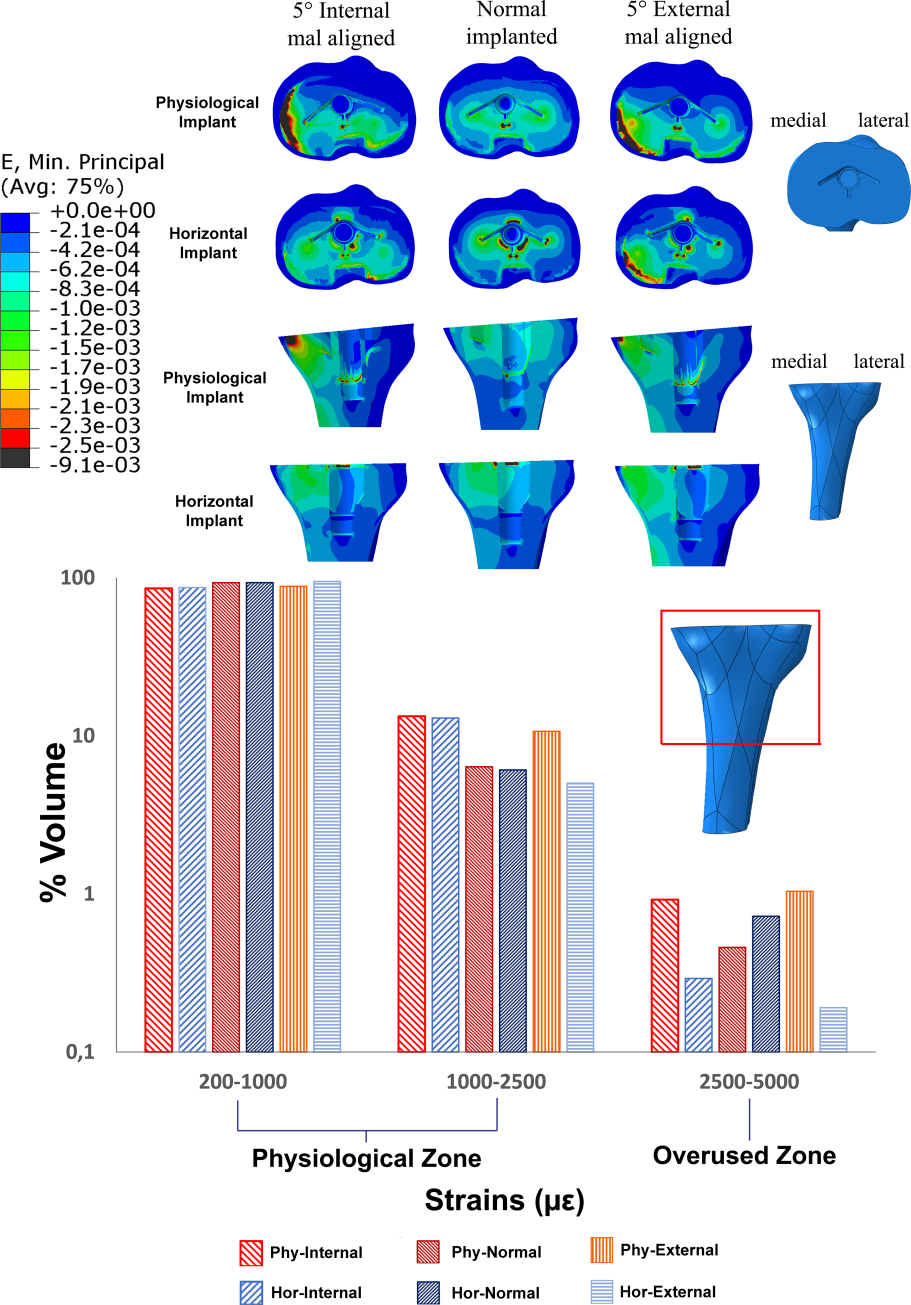
Strain distributions (60/40 medio-lateral load distribution) on the tibia component-tibia bone interface using of implants with a physiological joint line and a horizontal line. Strain distributions are represented in a normally positioned implant, as well as under 5 degrees of internal and external mal-rotation of the tibial component.

Considering the distribution of the strains in the selected volume during the mal-rotated conditions, a higher bone volume presented strains in the ranges of 1000–2500 με and 2500–5000 με in the physiological geometry (Fig 3).

## Discussion

The proper coronal alignment of the femoral and tibial components during TKA is matter of actual discussion, being the mechanically and kinematically aligning procedures the usual methods applied. Defenders of the mechanical alignment technique praise mostly the equal load distribution and apparent improvement of function [5–10], while for the kinematical alignment, the attempt to achieve an alignment similar to the preoperative state is highlighted [11, 18, 19]. Detractors focus on the alteration of the physiological joint line in the mechanical alignment [11–14] and the possible alteration of the medio-lateral load distribution during the kinematic alignment [18].

Considering that no clear consent exists among investigators, this study focused on the analysis of the influence of a prosthesis design with physiological joint line on the stress distribution on the PE-inlay as well as the strain distribution in the tibia component-tibia bone interface with particular focus on the axial mal-rotation of the tibia component, being this one of the most common causes of implant failure [38].

Previous FE studies focused on the axial mal-rotation have shown an increase in the contact stresses, particularly in high conformity designs [38] and that the greater the axial mal-rotation of the tibial component the higher the risk of edge contact and wear rate [25].

Our results from the analysis of the stresses on the PE-Inlay using loads measured during walking have shown an increase of the contact pressure in the physiological implant, which was clearly evident on the medial condyle and higher during the internal mal-rotated condition. However, considering the loading conditions applied and the changes during mal-rotation, neither of the determined contact stresses was greater than the 14.07 MPa yield stress of UHMWPE (with exception of the 70/30 medio-lateral load distribution during the 5° and 10° internal mal alignment), meaning that a local plastic deformation would not be expected in the conditions analysed.

Considering the results of the analysis of the strains on the tibia component-tibia bone interface, the strain values of the majority of the interface volume (>90%) analysed for the normal as well as for the mal-rotated conditions are in the physiological strain zone, which suggest no significant bone changes after implantation using both physiological and horizontal geometries. The physiological cut on the articular aspect of the tibia bone during the normal implantation showed apparently no detrimental effect on the load transfer compared to the horizontal implant. However, it seems to be more sensitive during the mal-rotated positions where a higher bone volume was more affected of higher strain values.

As it was already mentioned, the aim of the kinematical alignment is to place both femoral and tibial components in such a way that would emulate the preoperative knee joint alignment. Surgeons achieve this by resurfacing the knee joint [10], however special caution should be taken in order to not “exaggerate” the positioning of the components. Also, accurate kinematic alignment in varus is different from a malorientated varus results when mechanical alignment is the aim [4].

There are reports indicating that misalignment in the coronal plane during TKA is associated with reduced implant survival [26]. The survival ratio is supposed to be higher (90%) with a coronal implantation within 4° of the mechanical axis, whereas alignments higher than this would reduce the survival ratio to 70% [39–42]. A possible reason could be an abnormal load transfer across the knee joint to the tibia component-tibia bone interface.

Since the proposed physiological implant already comprises these physiological characteristics, it could lead to advantages during the implantation. Although these aspects are outside the scope of our study, the implantation of the physiological implant together with patient specific instrumentation, where the positioning of the components is based on bony landmarks established during preoperative planning [43], may be desirable in varus patients because a release of the collateral, posterior cruciate and retinacular ligaments will not be needed. This could lead to less operation trauma and faster recovery time guaranteeing also a physiological angulated bone cut.

Although the model was not experimentally validated, the loading conditions applied were directly calculated from *in vivo* measurements of a patient with a telemetric knee joint implant, which was key for the definition of a proper and physiological medio-lateral load distribution. The findings of our study should however be interpreted with caution. While realistic, only loads during level walking were applied and the changing of loading conditions during flexion was not considered and should be part of future analysis. Also, future *in vivo* analyses of the knee joint kinematics after physiological TKA using fluoroscopy could open new perspectives in the understanding of the influence of such adapted geometries.

Moreover, additional factors such as patients BMI, level of activity, medical comorbidities, response to joint surgery as well as surgeon skill and experience should also be considered in knee joint kinematic analyses in order to catalogue an implantation as successful.
